# Transcriptome analysis of HTLV-1-infected cells in comparison to the normal and Tax-expressing T cells

**DOI:** 10.1186/1742-4690-8-S1-A123

**Published:** 2011-06-06

**Authors:** Shruti Singh, Kevin Quann, Jordana Coelho-dos-Reis, Zafar K Khan, Pooja Jain

**Affiliations:** 1 Department of Microbiology and Immunology, and the Drexel Institute for Biotechnology and Virology Research, Drexel University College of Medicine, Doylestown, PA, 18902, USA

## 

The transactivator protein of HTLV, Tax, is required for the activity of viral promoter LTR (long terminal repeat) and is capable of regulating both virus and host transcription. Tax is known to interact with several cellular factors including CREB/ATF, NF-κB and those involved in chromatin remodeling, cell cycle and DNA damage/repair. However, the process of Tax-mediated transactivation of chromosomally integrated LTR is not fully understood. Therefore, we have performed an extensive Protein/DNA transcription factor array (PD array I, Panomics) analysis on the HTLV-1 infected cells (MT-2) in comparison to the Tax-expressing cells (C8166) and normal T cells (Jurkat). Besides known factors, significant changes were observed in several new nuclear and cytoplasmic factors such as RxR, AP-1, AP-2, in both MT-2 and C8166 cells as compared to Jurkat indicating an overlap with viral replication and Tax-activity. Interestingly, MT-2 cells demonstrated the down-regulation of majority of the factors analyzed while several nuclear (NF-E1, NF-E2, TFIID) and cytoplasmic (MEF-1, RAR) factors were upregulated in C8166 suggesting Tax activity independent of viral replication. Overall, this data suggest that more scrutiny on nuclear and cytoplasmic factors associated with Tax transactivation process is needed to clearly elucidate the mechanism underlying adult-T-cell leukemia and tropical spastic paraparesis associated with HTLV-1 infection.

